# Personalized Radiation Attenuating Materials for Gastrointestinal Mucosal Protection

**DOI:** 10.1002/advs.202100510

**Published:** 2021-04-27

**Authors:** James D. Byrne, Cameron C. Young, Jacqueline N. Chu, Jennifer Pursley, Mu Xian Chen, Adam J. Wentworth, Annie Feng, Ameya R. Kirtane, Kyla A. Remillard, Cindy I. Hancox, Mandar S. Bhagwat, Nicole Machado, Tiffany Hua, Siddartha M. Tamang, Joy E. Collins, Keiko Ishida, Alison Hayward, Sarah L. Becker, Samantha K. Edgington, Jonathan D. Schoenfeld, William R. Jeck, Chin Hur, Giovanni Traverso

**Affiliations:** ^1^ Division of Gastroenterology Brigham and Women's Hospital Harvard Medical School 75 Francis St. Boston MA 02115 USA; ^2^ Harvard Radiation Oncology Program 55 Fruit Street Boston MA 02114 USA; ^3^ David H. Koch Institute for Integrative Cancer Research Massachusetts Institute of Technology 500 Main St Building 76 Cambridge MA 02142 USA; ^4^ Department of Mechanical Engineering Massachusetts Institute of Technology 77 Massachusetts Ave Cambridge MA 02139 USA; ^5^ Department of Radiation Oncology Dana‐Farber Cancer Institute/Brigham and Women's Hospital 44 Binney St. Boston MA 02115 USA; ^6^ Division of Gastroenterology Massachusetts General Hospital 55 Fruit St. Boston MA 02114 USA; ^7^ Division of Medical Physics Department of Radiation Oncology Massachusetts General Hospital 450 Brookline Avenue Boston MA 02115 USA; ^8^ Division of Comparative Medicine Massachusetts Institute of Technology Building 16‐825, 77 Massachusetts Ave Cambridge MA 02139 USA; ^9^ Department of Pathology Duke University Durham NC 27710 USA; ^10^ Department of Medicine Columbia University Medical Center 622 West 168th Street, PH 9‐105 New York NY 10032 USA; ^11^ Department of Epidemiology Mailman School of Public Health and Herbert Irving Comprehensive Cancer Center Columbia University Medical Center 722 West 168th St. New York NY 10032 USA

**Keywords:** 3D printing, dosimetric analysis, radiation attenuation, radiation‐induced mucositis, radiation proctitis, radioprotective devices

## Abstract

Cancer patients undergoing therapeutic radiation routinely develop injury of the adjacent gastrointestinal (GI) tract mucosa due to treatment. To reduce radiation dose to critical GI structures including the rectum and oral mucosa, 3D‐printed GI radioprotective devices composed of high‐Z materials are generated from patient CT scans. In a radiation proctitis rat model, a significant reduction in crypt injury is demonstrated with the device compared to without (*p* < 0.0087). Optimal device placement for radiation attenuation is further confirmed in a swine model. Dosimetric modeling in oral cavity cancer patients demonstrates a 30% radiation dose reduction to the normal buccal mucosa and a 15.2% dose reduction in the rectum for prostate cancer patients with the radioprotectant material in place compared to without. Finally, it is found that the rectal radioprotectant device is more cost‐effective compared to a hydrogel rectal spacer. Taken together, these data suggest that personalized radioprotectant devices may be used to reduce GI tissue injury in cancer patients undergoing therapeutic radiation.

## Graphical abstract

One of the most common side effects of radiation therapy is injury to the gastrointestinal (GI) tract. To reduce radiation‐induced injury, personalized 3D‐printed radioprotective devices made of high‐Z materials are developed that are shown to reduce radiation injury in two animal models, improve clinically significant dosimetry in at‐risk GI tissue in human cancer patients, and are more cost‐effective compared to standard‐of‐care therapy.

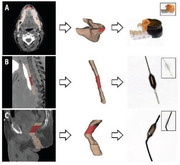

## 1 Introduction

Radiation attenuating materials are integral to the safety of all diagnostic radiology and radiation oncology practices worldwide. These materials traditionally have a high atomic number (high‐Z), such as lead, and are capable of reducing ionizing radiation exposure.^[^ ^1^ ^]^ Despite their ability to mitigate radiation exposure, these materials have not been directly integrated into patient treatments due to the inability to rapidly generate personalized attenuating devices.

Most cancer patients undergoing therapeutic radiation will develop normal tissue injury as a result of treatment.^[^ ^2^ ^]^ The toxicities resulting from radiation‐induced normal tissue injury are dependent upon the location of treatment, with the most common toxicities involving the oral cavity and gastrointestinal (GI) tract in the forms of oral mucositis, esophagitis, and proctitis.^[^ ^3^, ^4^, ^5^, ^6^, ^7^ ^]^ It is estimated that radiation‐induced GI toxicities occur in over 200 000 patients in the United States every year (Figure S1, Supporting Information).^[^ ^5^, ^6^, ^8^, ^9^, ^10^ ^]^ This normal tissue injury may lead to severe morbidity and, ultimately, treatment breaks or discontinuation that adversely impact tumor cure rates.^[^ ^11^, ^12^ ^]^ Currently, attempts to reduce radiation‐induced side effects such as physical spacers, shielding, and treatments for radiation‐induced mucositis have many limitations in protecting normal tissues, including concerns regarding diminishing intended tumor treatment, dependency on user experience, and additional side effects.^[^ ^13^, ^14^, ^15^, ^16^ ^]^ New methods for radiation protection are needed to reduce morbidity.

Here, we describe the development and feasibility of a new class of personalized 3D‐printed radioprotectant devices with integrated attenuating materials to prevent radiation‐induced toxicities in cancer patients. We conceptualize that due to their personalized manufacturing, these devices can enable improved dosimetric advantage and compliance compared to generic systems. These devices have the potential to shift the paradigm of clinical management of patients receiving radiation therapy for cancer; by reducing radiation‐associated morbidity and therefore improving treatment adherence, they have the potential to improve survival.

## 2 Results

### 2.1 Fabrication of Personalized 3D‐Printed Radioprotectant Devices with Rationally Placed Attenuating Agents

**Figure 1:**
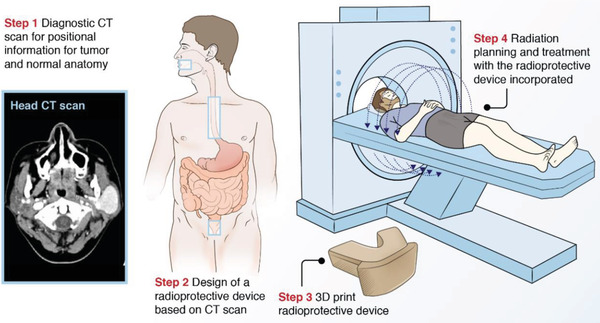
Clinical workflow for integrating personalized radioprotectant devices in radiation treatments.

**Figure** **1** depicts the clinical workflow for generating personalized 3D‐printed radioprotective devices according to the specific organs at risk for radiation‐induced injury. Cancer staging scans are routinely used for radiation treatment planning and can be easily integrated into device development. The organs at risk for radiation‐induced injury vary according to anatomical location of treatment. Based upon the burden of radiation‐induced GI toxicities in these areas, we wanted to focus on the following organs at risk: buccal mucosa in oral cavity cancer patients, esophagus in lung cancer patients, and rectum in prostate cancer patients.^[^ ^5^, ^6^, ^17^ ^]^

**Figure 2:**
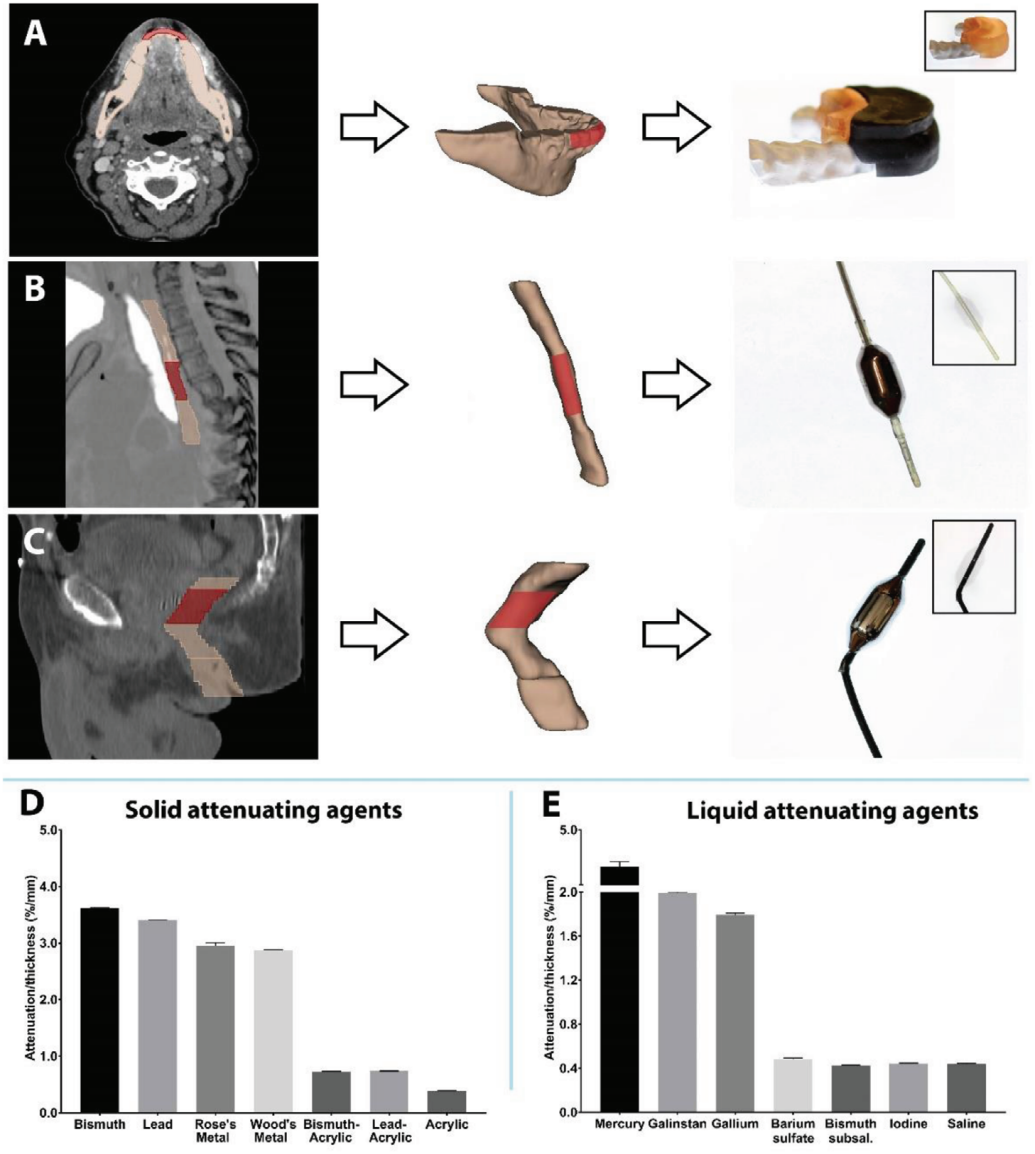
Personalized 3D‐printed devices used for radioprotection of various anatomical sites at high risk for radiation toxicity. Prototypes of A) intra‐oral device, B) esophageal device, and C) rectal device generated from patient data. The area of interest for protection is highlighted in red. Radiation attenuation with D) solid high‐Z materials and E) liquid high‐Z materials.

Using 3D slicer, the organs at risk for radiation‐induced damage were contoured on patient diagnostic DICOM scans (**Figure** **2**A). The contours were generated into 3D models (Figure 2B), and personalized 3D‐printed devices with incorporated attenuating materials were generated to fit these models (Figure 2C).

For each of the sites, patient anatomy was shown to vary tremendously (Table S2, Supporting Information). The ability to 3D print these attenuating materials aids in the expedient fabrication of customized devices.^[^ ^18^, ^19^ ^]^ Personalizing each device may improve patient comfort, as well as the degree of radiation protection, by positioning the device near the high radiation dose regions while not impacting the effectiveness of the treatment. For intra‐oral devices, we attempted to adhere to several key design traits, including rounding at buccal peripheries and reducing palatal extension and sizes (≤10 mm) to improve patient comfort and adherence.^[^ ^20^ ^]^ Determining the location of the radiation‐attenuating agent requires input from both the radiation oncologist and dosimetrist to ensure the correct areas are protected.

An additional component of the device necessary for compatibility with image‐guided radiation therapy (IGRT) was the ability to replace or remove the attenuating material to reduce image artifacts on preradiation treatment imaging and, more appropriately, represent anatomical distortion from the device. The balloon catheters may be filled with saline for the pretreatment imaging scans and then replaced with the high‐Z material when the patient has been correctly positioned for treatment. Similarly, intra‐oral devices can be generated with non‐attenuating materials that can be replaced following IGRT.

### 2.2 Characterization of Radiation Attenuation in High‐Z Materials

To determine the materials that would offer the greatest degree of shielding, we characterized the radiation attenuation of numerous high‐Z materials based on mass attenuation coefficients reported by National Institute of Standards and Technology (Figure 2D,E).^[^ ^21^ ^]^ The attenuation studies performed herein refer to relative attenuation values as we did not use a narrow‐beam setup to eliminate any low‐energy scattered photons. Methods for testing can be found in the Supporting Information. The materials tested included elements, alloys, and composites, all of which have been previously incorporated into 3D‐printed devices.^[^ ^22^ ^]^ To quantify the degree of radiation attenuation, the materials were formed into blocks or filled into 25 cm^2^ flasks larger than a Farmer chamber and subsequently exposed to 6 MV photon radiation from a linear accelerator. Elemental materials demonstrated greater radiation attenuation compared to alloys and composites (Figure 2D,E). Elemental bismuth and lead had approximately three times greater attenuation compared to their composite counterparts, which had been loaded with 50% lead and bismuth particles in an acrylic resin. Among the liquid attenuating agents, mercury had greater attenuation compared to all other liquid materials, over twice that of the second most attenuating material, Galinstan.

### 2.3 In Vivo Evaluation of Radioprotectant Devices

Having established the radiation attenuation for these materials, we next tested the in vivo application of our radioprotective devices using single‐dose radiation‐induced oral mucositis and proctitis rat models.^[^ ^23^, ^24^, ^25^ ^]^ The radioprotective devices informed by computed tomography (CT) scans were developed according to the clinical workflow (Figure 1). The attenuating devices were designed to protect approximately half of the area that is at risk for normal tissue injury. Seven out of seven control animals treated with radiation to the oral cavity had gross ulcerations on their tongue compared to zero of seven animals with the radioprotective device in place. Additionally, a laboratory veterinarian performed colonoscopies at day 8 on a control animal and an experimental animal treated with radiation to the rectum to evaluate for onset of proctitis; increased erythema was noted in the rectum of the control animal compared to the experimental animal.

**Figure 3:**
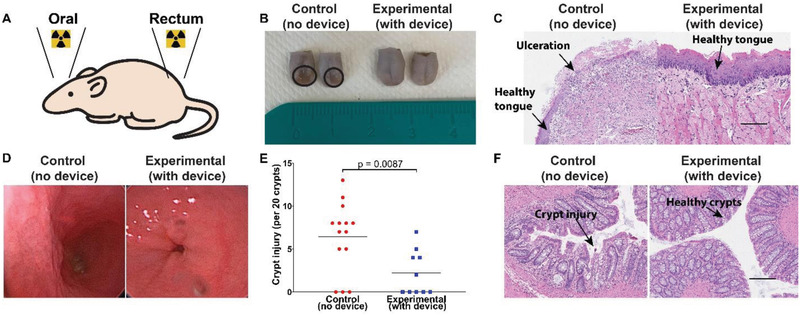
Radioprotective effect of intra‐oral and rectal devices in rats. A) Anatomical location of radiation treatment. B) Gross tissue evaluation at 9 d post‐treatment showcasing tongue ulceration in the control group (no device) compared to the normal, healthy appearing tongues in the experimental group (with radioprotective device). C) Histological H&E staining of representative tissues demonstrating ulceration in the control group compared to healthy appearing tongue tissue in the experimental group. Scale bar is 100 µm. D) Representative colonoscopic images from rats treated with and without the device. There was more erythema noted in the rectum of rats treated without the device. E) Quantitation of the crypt injury as defined by crypt epithelial flattening, intraepithelial or luminal inflammation, or crypt drop out and quantified as the greatest number of injured or absent crypts per 20 consecutive crypts. *P*‐value was determined by unpaired *t*‐test. F) Histological H&E staining of representative tissues demonstrating crypt injury in the control group compared to healthy crypts in the experiment group. Scale bar is 100 µm.

Histological analysis of oral tongue revealed extensive ulceration (**Figure** **3**C) on the dorsal surface of the tongue in 7/7 control animals (up to 4.0 mm in one animal by histologic assessment), with nuclear atypia in the stroma also noted. Of the animals treated with the radioprotective device in place, there was one punctate ulcer in 1/7 animals (largest dimension 0.75 mm), which was a significant improvement compared to the control group. Radiation‐induced rectal injury resulted in histologically identifiable crypt injury, defined here as crypt epithelial flattening, intraepithelial or luminal inflammation, or crypt drop out and quantified as the greatest number of injured or absent crypts per 20 consecutive crypts. Control animals experienced significantly greater crypt injury compared to animals treated with the radioprotective device in place, as seen in Figure 3E,F.

**Figure 4:**
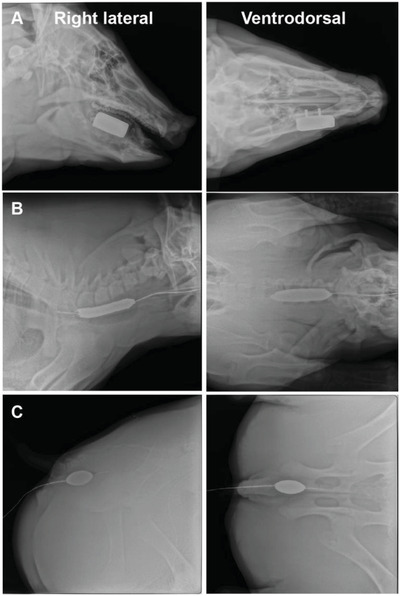
Positioning of intra‐oral, esophageal, and rectal radioprotectant devices in swine. Right lateral (left) and ventrodorsal (right) radiographs of devices in A) oral cavity, B) esophagus, and C) rectum.

Next, we designed and generated swine intra‐oral, esophageal, and rectal devices based upon CT imaging. The placement of the devices was evaluated in three anesthetized Yorkshire swine (weights ranging between 45 and 65 kg), as demonstrated in **Figure** **4**. The intra‐oral devices were designed for protection of buccal mucosa with the attenuating material located on the lateral aspect of the teeth and jaw. The facile placement of these devices in the large animal model indicates anticipated success in translation to the clinical setting.

### 2.4 Dosimetric Evaluation in Patients

**Figure 5:**
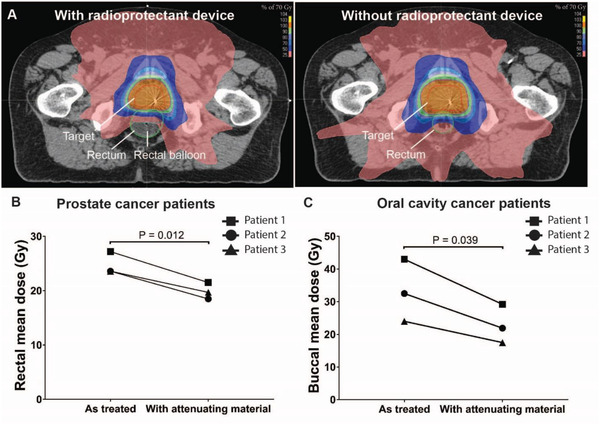
Dosimetric modeling of radioprotectant devices in patients. A) Axial CT images of radiation plan of a prostate cancer patient with a radioprotectant device compared to without a radioprotectant device (as treated) showcasing the impact of the device on reducing radiation exposure to the rectum. Comparison of dosimetric plan with or without attenuating material in B) prostate cancer patients (*n* = 3) and C) oral cavity cancer patients (*n* = 3). Mean doses were calculated as the average of the dose to each voxel contained within the organ; voxels of 2 mm × 2 mm in *X* and *Y*, and 2.5 mm in the *Z* direction. *P*‐value was determined by paired two‐sample *t*‐test.

To determine the degree of radioprotection expected in patients treated with the current standard‐of‐care radiation delivery method, known as intensity‐modulated radiation therapy (IMRT), we modeled the attenuating devices in our radiation treatment planning software in both prostate cancer patients and head and neck cancer patients. Many prostate cancer patients have been treated with rectal balloons filled with saline to protect the posterior rectal wall and minimize day‐to‐day variability of the prostate and rectum.^[^ ^26^ ^]^ The attenuating devices were designed to protect approximately half of the at‐risk normal tissue. **Figure** **5**A showcases a patient with and without the rectal balloon and the dosimetric differences when the balloon is filled with liquid attenuating material having the density of mercury. Next, we evaluated the dosimetric advantage of a liquid attenuating material in the rectal balloon in three prostate cancer patients treated with radiation compared to filling the balloon with saline. The patients with the liquid attenuating agent in the rectal balloon were found to have 15.2% dose reduction compared to those without the attenuating agents (Figure 5B). Figure S5 (Supporting Information) demonstrates a dosimetric comparison of the primary target volume and organs‐at‐risk with and without the radioprotective rectal balloon. For our head and neck cancer patients, we contoured in model devices to protect the buccal mucosa and placed the attenuating material in a location of high radiation dose exposure and minimal entrance radiation beams. To account for back scatter from the attenuating material, we added a 3 mm isodense film on the buccal side of the device. A 30% dose reduction was quantified at the buccal mucosa adjacent to the device as a result of beam attenuation and tissue displacement (Figure 5C). Dose metrics can be found in Tables S3 and S4 (Supporting Information).

### 2.5 Cost‐Effectiveness Analysis

**Figure 6:**
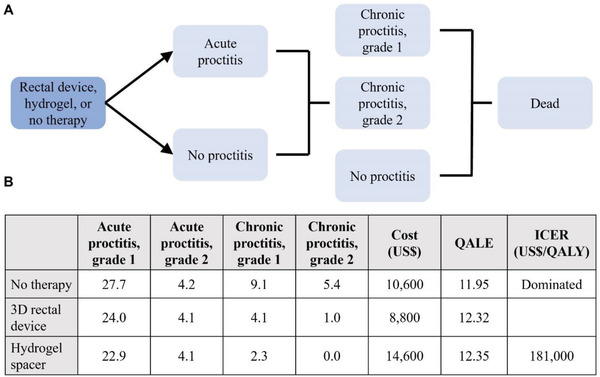
Cost‐effective analysis of the 3D‐printed rectal device. A) Schematic for the cost‐effective analysis comparing our 3D‐printed rectal radioprotectant device, the hydrogel spacer, and no prophylactic therapy for patients with localized prostate cancer undergoing radiation therapy. B) Results of the base case analysis showcasing our 3D‐printed rectal device was the cost‐effective strategy. The hydrogel spacer was not cost‐effective compared to the rectal device as it was too expensive with an ICER of $181 000/QALY (more than the willingness‐to‐pay threshold of $100000/QALY). The no prophylactic therapy strategy was dominated (less effective and more costly) than our radioprotectant rectal device. QALE, quality‐adjusted life expectancy; ICER, incremental cost‐effectiveness ratio; QALY, quality‐adjusted life year.

To estimate the potential clinical utility and cost‐effectiveness of the radioprotective devices, we developed a decision analytic Markov model to compare the 3D‐printed rectal radioprotectant device for prevention of radiation proctitis to the hydrogel spacer (SpacerOAR) and to no prophylactic treatment in patients receiving radiation therapy for localized prostate cancer (**Figure** **6**A). Using the clinical dosimetric evaluation of our rectal radioprotectant devices, we estimated the rectal device to be at least 75% as effective in rectal sparing as the hydrogel spacer based upon a similar reduction in median volume of rectum treated to ≥70 Gy (Table S3, Supporting Information) compared to published historical hydrogel spacer controls.^[^ ^27^ ^]^ We used the more conservative estimate of 75% efficacy for the base case in our cost‐effective analysis and found that the rectal radioprotectant device was the cost‐effective strategy (Figure 6B; Figure S6 and Table S5, Supporting Information). In addition, the rectal device was cost saving (both more effective and less costly) compared to no prophylactic therapy by avoiding costs incurred by chronic radiation proctitis.

We conducted a sensitivity analysis in which the rectal device had the same efficacy as the hydrogel rectal spacer (Table S5, Supporting Information). At the same degree of efficacy, the rectal device would be both more effective (due to avoidance of a procedure required for hydrogel implantation) and less costly than the hydrogel spacer; in other words, the rectal device would be cost saving compared to the hydrogel spacer. As in the base case, the rectal device is also more effective and less costly than no prophylactic therapy.

Additional sensitivity analyses for key model inputs or parameters were performed and are summarized in Figure S2 (Supporting Information). The model results were sensitive (the optimal strategy changed from rectal device to hydrogel spacer) to the following threshold values: starting age younger than 63, disutility of hydrogel placement better than −0.5, disutility of the rectal device worse than −0.03, and probability of adverse event from the hydrogel spacer placement of greater than 0.025.

## 3 Discussion

Sparing normal tissue toxicity by reducing radiation dose exposure is one of the primary goals of radiation oncology. The advent of radiation technologies, including IMRT, daily cone‐beam computed tomography, proton therapy, and better patient immobilization have had profound benefit for cancer treatment. However, patients continue to experience severe oral cavity and GI toxicities that result in morbidity and, at times, mortality.^[^ ^2^ ^]^ Shown here, our radiation‐attenuating devices reduced oral mucositis and proctitis in animals and modeling showed dose sparing in human simulations. We also apply the novel concept of personalizing and 3D‐printing systems to mitigate radiation treatment toxicity and developed a standardized process for contouring CT scans into organ models to generate 3D‐printed radioprotective devices. Verified in both small and large animal models as well as human simulations, our results support the feasibility of personalized devices for reduction of radiation dose and associated side effects through displacement and attenuation of the radiation dose. This strategy can be easily integrated into the clinical workflow of cancer therapy, as represented in Figure 1, and as shown in our cost‐effective analysis, could provide significant cost saving compared to no prophylactic therapy in the form of reduced hospitalizations, re‐admissions, emergency room visits, and interventional treatments, while improving patient comfort and outcomes. While we recognize that many of the attenuating materials used in our studies are toxic, several devices that have such materials have been successfully translated to human applications, such as dental amalgam, sphygmomanometers, and thermometers.^[^ ^28^ ^]^ Furthermore, initial cytotoxicity testing of one of our silicone‐coated lead intra‐oral radioprotectant devices demonstrated no cytotoxicity compared to controls (Figure S7, Supporting Information) under testing adhering to the Food and Drug Administration guidance for temporary placement of mucosal devices.^[^ ^29^ ^]^ Other clinically used radioprotectant devices also incorporate attenuating materials that may be toxic (Table S6, Supporting Information).

These are initial proof‐of‐concept studies and additional investigation will be required for full clinical translation. Future efforts toward human translation will have to expand our experiments to higher energy sources delivered with linear accelerators and fractionated dosing as we conducted the rodent studies with single treatments with a cesium irradiator with an energy source of 0.662 MV, whereas linear accelerators deliver high energies for clinical treatments.^[^ ^30^ ^]^ Additional toxicity evaluation of the devices, including liquid attenuating agents, is needed to ensure safety of these devices. Investigation into the impact of the attenuating material on image artifacts and anatomical distortion compared to saline or non‐attenuating materials incorporated during IGRT will be necessary. Finally, given the small sample size of our dosimetric studies, further investigation in larger cohorts is needed to validate these approaches. However, these data suggest personalized 3D‐printed radioprotectant devices may have great potential to reduce radiation toxicity in clinical settings where radiation is used, including neoadjuvant and adjuvant therapy, curative treatment, and palliative treatments. This personalized approach could be applicable to a variety of cancers that respond to radiation therapy, including head and neck, lung, prostate, anal, skin, and gynecological cancers, sarcomas, and lymphomas.

## 4 Experimental Section

##### Device Design

It was sought to create a physical barrier to attenuate RT dose which could be placed along the GI tract with the goal of reducing dose to the surrounding epithelium and soft tissue structures using a 3D‐printed device. Various iterations of this device were designed using opensource CAD design software to create design suited to protect either the oral cavity, the esophagus, or the prostate via the rectum. The form factor of the radioprotective device was influenced by the patient's anatomy and the organs at risk for radiation‐induced damage. For oral cavity cancer patients where buccal tissue is commonly injured, an intra‐oral device was generated, whereas for lung and prostate cancer patients who are at risk for damage of the esophagus and rectum, respectively, balloon catheters were developed similar to already established devices, such as Minnesota or Blakemore tubing, esophageal dilators, and rectal balloons.^[^ ^30^, ^31^ ^]^ Esophageal dilators are used frequently in certain patients with esophageal strictures, and thus, a balloon catheter strategy was thought to be reproducible and feasible.^[^ ^32^, ^33^ ^]^ The degree of radiation attenuation of solid and liquid materials was next tested. The radiation attenuation is a result of the interaction between high energy photons/gamma rays with the high‐Z materials resulting in loss of energy. The material composition directly affects radiation attenuation, but the form of elemental materials (solid vs liquid) has minimal effect on radiation attenuation per NIST standards as there is no significant change in density from solid to liquid form.^[^ ^21^ ^]^ Pure elemental bismuth and lead 1 cm thick sheets (McMaster‐Carr) were trimmed to 6 × 6 × 1 cm in dimension. The Rose's metal (Rotometals, 50% bismuth, 25% lead and 25% tin, melting point 94 Celsius) and Wood's metal ingots (Rotometals, 50% bismuth, 25% lead, 12.5% tin, and 12.5% cadmium, melting point 70 °C) were melted into 6 × 6 × 1 cm silicone molds for size matching. Finally, lead particles (Sigma) and bismuth nanoparticles (American Elements) were loaded to 50 wt% in a photocurable acrylic resin (Formlabs) and placed in the silicone mold prior to curing with a UV lamp. Liquid materials were placed into 25 cm^2^ flasks that yielded a thickness of 1 cm. Pure elemental mercury and gallium (Sigma) were used; gallium was heated to 40 °C to maintain liquid consistent throughout the experiment. Galinstan (Sigma, 68.5% gallium, 21.5% indium, and 10.0% tin), barium sulfate (READI‐CAT, 0.3% wt./vol.), bismuth subsalicylate (Pepto Bismol, 1.75% wt./vol.), and iodine (Sigma, 0.5 m) were used as provided. The thickness of each side of the flask was ≈1 mm, to minimize the impact of the flask material on attenuation. Prior to testing the materials, baseline radiation measurements were performed by administering 100 monitor units of a 6 MV photon beam over a 10 × 10 cm square at 100 cm from the Farmer Chamber. 3 cm of solid water was placed on top of 2 cm Farmer chamber holder on top of 9 cm solid water. Subsequently, materials were placed directly on top of the solid water over the Farmer chamber to measure the degree of attenuation, and a total of six runs were performed per sample. The samples were normalized to the thickness of the material. All testing was performed at Massachusetts General Hospital. Each design was then tested in vivo, first in rats to confirm efficacy of radiation attenuation and then in swine to confirm device placement in a larger animal system to ensure translation of device to humans. Retrospective analysis of human DICOM scans was used to evaluate dosimetric advantage of the device in oral and prostate cancer patients.

##### Rats

All procedures were approved by the Committee on Animal Care at Massachusetts Institute of Technology (MIT) (Protocol No. 0519‐023‐22) before initiation and all procedures described herein conform to the Committee's regulatory standards. The rats used in this study were eight‐week‐old female Sprague‐Dawley rats. Experiments were conducted at MIT Koch Institute animal facilities after one week of acclimation. Animals were randomized into experimental (*n* = 7, received radioprotectant device) and control (*n* = 7, did not receive the radioprotective device).

CT scans of Sprague‐Dawley rats were obtained from the Koch Institute imaging facility. Models of the oral cavity were 3D‐printed on a Formlabs printer, and a high temperature silicone mold was generated from the 3D‐printed model. Elemental lead was melted and cast into this mold to generate an oral cavity replica. To generate rectal devices, hollow tubes were designed in SolidWorks to match the radius and length of the organ of interest and then printed on a Lulzbot TAZ 5 FDM printer. Instead of balloon catheters, solid radioprotectants were used due to the ease of application and testing in the rat model. Furthermore, the attenuating material chosen for these studies was elemental lead because of the degree of attenuation and ease of production. Figure S3 (Supporting Information) demonstrates example devices used in these rats, and Figure S7 (Supporting Information) demonstrates the safety of encapsulating the lead‐containing devices in polydimethylsiloxane (PDMS).

Investigators and animal technicians could not be blinded during the conduct of the experiment due to the device placement and were not blinded during outcome assessment and data analysis. The clinical pathologist was blinded before and during histological analysis. No inclusion or exclusion criteria were set a priori. No animals were excluded from analysis. Prior to any treatments, dose calculations for custom‐made lead shielding for efficacy studies in rats were performed using optically stimulated luminescent dosimeters to verify dose. An output factor of 0.71 was calculated and applied to the animal studies. Rats were anesthetized using 1–3% isoflurane, and the rats were placed in a custom‐made polycarbonate holder that allowed for pulse oximetry and heart rate monitoring. The holder was set into a custom‐made lead shielding device. A 2 cm collimator was opened in the lead shielding, and the animals were exposed to 18 Gy at the anatomical location of interest—oral cavity or rectum—from a single cesium source from a Gammacell 40 Extractor irradiator (Figure S4, Supporting Information). After completion of radiation, the animals were evaluated twice daily. Any animal that exhibited signs of morbidity or weight loss was administered buprenorphine. The animals were euthanized 9 d after completion of radiation, and tissue was formalin fixed prior to histologic evaluation with H&E staining at the MIT Koch Institute histology core. Outcome measures were defined as extent of tongue ulceration, extent of colonic erythema on colonoscopy, and extent of radiation‐induced rectal injury defined as crypt epithelial flattening, intraepithelial or luminal inflammation, or crypt drop out and quantified as the greatest number of injured or absent crypts per 20 consecutive crypts.

##### Swine

All procedures were approved by the Committee on Animal Care at MIT (Protocol No. 0519‐023‐22) before initiation and all procedures described herein conform to the Committee's regulatory standards. The swine used in this study were healthy female Yorkshire pigs between 45 and 65 kg Yorkshire swine. Experiments were conducted at MIT large animal facilities. Animals (*n* = 3) were not randomized and all received the device.

CT scans of Yorkshire pigs were obtained from the Whitaker College Imaging Facility. Models of the oral cavity were 3D‐printed on a Formlabs printer, and a high temperature silicone mold was generated from the 3D‐printed model. Elemental lead was melted and cast into this mold to generate an oral cavity replica. To generate rectal devices, hollow tubes were designed in SolidWorks to match the radius and length of the organ of interest and then printed on a Lulzbot TAZ 5 FDM printer.

The placement of the devices was evaluated in three Yorkshire swine. An output factor of 0.71, calculated from efficacy studies in rats of custom‐made lead shielding, was applied. Investigators and animal technicians could not be blinded during the conduct of the experiment due to the device placement and were not blinded during outcome assessment and data analysis. No animals were excluded from analysis.

During the device placement procedure, swine were anesthetized, and devices were placed in the oral, esophageal, or rectal location. For ease of placement of the esophageal device, the device was thread through a catheter sheath to the proximal‐to‐mid esophagus and then filled with a liquid attenuating material prior to radiographs. Finally, the rectal device was placed into the distal rectum and subsequently filled with the liquid attenuating material prior to radiography. Upon confirmation of device placement, the device was filled with a liquid attenuating agent.

##### Human Subjects

Institutional Review Board (IRB) approval was obtained prior to any work (Partners IRB 2018P002468). Patients with oral cavity, lung, and three with prostate cancers were selected for design of the devices. Patients with oral cavity and prostate cancers (*n* = 3 per cancer type) were used for dosimetric modeling.

Patient diagnostic was obtained by using Digital Imaging and Communications in Medicine (DICOM) scans, and using 3D Slicer, the volumes of interest were contoured, and the personalized mouthguard designed in Meshmixer. Following printing on the Formlabs Form 2 3D printer, the devices were manually post‐processed following the 3D printer guidelines.

Three previously treated oral cavity cancer patients were identified for dosimetric modeling in Eclipse version 15.6. Certified medical dosimetrists created the dosimetric plans used in these studies. The oral cavity device is a shield filled with high‐Z material placed within the oral cavity proximal to the buccal mucosa and designed based on the patient's specific anatomy with the use of CAD design. The locations for the devices were rationally chosen to be (1) in an area of dose spill‐off outside of the target regions and (2) < 2 entrance beams going through the device. A multicomponent device was contoured in the specific location; the components of the device included an internal 7 mm of high‐Z material with density corrected up to 10 g cm^−3^ and an external 3 mm polymeric material with density of 1 g cm^−3^ to prevent dose scatter. It was shown that at 2 mm the contribution from back scatter is less than 2% and at 1 mm it is less than 4% for high‐Z materials.^[^ ^34^, ^35^ ^]^ The tissue contours were adjusted to account for displacement of the tissue. Buccal tissue was contoured to quantify dose. Each patient's case underwent re‐planning to account for the device. The buccal tissue dose was compared between plans.

Three previously treated prostate cancer patients with rectal balloons were selected for dosimetric modeling in Raystation version 5.0, which uses collapsed cone superposition‐convolution algorithm. Certified medical dosimetrists created the dosimetric plans used. Grid size was 0.2 × 0.25 × 0.2 cm.  IMRT and saline‐filled rectal balloons were used in the treatment of these patients. The rectal balloon was contoured, and the density was adjusted to match the density of mercury. Each patient's case underwent re‐planning, and the rectal dosing was compared between plans. This modeling aimed to mimic the clinical benefit of the rectal device, which is a shield filled with high‐Z material placed within the rectum to protect the prostate from acute radiation exposure during treatment.

##### Cost‐Effectiveness Analysis

A Markov‐state transition model was developed in TreeAge Pro (TreeAge, Williamstown, MA) to compare the effectiveness and cost‐effectiveness of three prevention strategies for radiation proctitis: no prophylactic therapy, hydrogel rectal spacer, and the personalized 3D‐printed radioprotectant rectal device. The model simulated a hypothetical cohort of 67‐year‐old men who received radiation therapy for T1 or T2 prostate cancer based on patients enrolled in a SpacerOAR trial.^[^ ^14^, ^26^ ^]^ All patients began well other than their cancer for which they were receiving radiation therapy and were followed until death. In all treatment options, the patients could remain well without proctitis, develop acute radiation proctitis, develop grade 1 chronic radiation proctitis, develop grade 2+ chronic radiation proctitis, or die of age‐ and sex‐related mortality. Cancer‐related mortality for early‐stage prostate cancer approaches zero; therefore, patients were assumed to have no additional cancer‐specific mortality.^[^ ^36^, ^37^ ^]^

All parameters for the hydrogel rectal spacer and no shield treatment arms were estimated from SpacerOAR trial and other published literature (Table S1, Supporting Information).^[^ ^38^, ^39^, ^40^, ^41^, ^42^, ^43^, ^44^, ^45^, ^46^, ^47^, ^48^, ^49^, ^50^, ^51^, ^52^, ^53^, ^54^, ^55^, ^56^, ^57^, ^58^ ^]^ The model was partially based off a previously published model.^[^ ^44^ ^]^ Base‐case probabilities of developing radiation proctitis in the radioprotective rectal device treatment arm were estimated assuming the personalized radioprotectant rectal device would be 75% as effective as the hydrogel spacer. This estimate is based upon similar reduction in rectal V70 (>25%) compared to the hydrogel spacer; however, the rectal device has a relative lack of protection of the anterior wall of the rectum.^[^ ^30^ ^]^ Other parameters for the rectal device were estimated from the literature (Table S1, Supporting Information). Sensitivity analyses were performed for key parameters in the model (Figure S2, Supporting Information).

##### Statistics

Statistical analysis was performed using SAS v9.3 software, and all data are expressed as means ± SD. Copyright © 2019 SAS Institute Inc. SAS and all other SAS Institute Inc. product or service names are registered trademarks or trademarks of SAS Institute Inc., Cary, NC. All sample sizes can be found in Section 2 and refer to number of animals and patients. Unpaired *t*‐tests were used to compare treatment groups for rat studies. Paired *t*‐tests were used for clinical dosimetric studies. *P* value of < 0.05 was considered significant.

## Conflict of Interest

The authors declare no conflict of interest related to this work. Complete details of all relationships for profit and not for profit for G.T. can found at the following link: https://www.dropbox.com/sh/szi7vnr4a2ajb56/AABs5N5i0q9AfT1IqIJAE‐T5a?dl=0.

## Supporting information

Supporting Information

## Data Availability

Research data are not shared.
