# Supplementary material for: Personalized Radiation Attenuating Materials for Gastrointestinal Mucosal Protection
Source: Adv Sci (Weinh). 2021 Apr 27;8(12):2100510. doi: 10.1002/advs.202100510 (PMC8224439; doi:10.1002/advs.202100510)
Supplement: Supplementary file 1 — Supporting Information [file ADVS-8-2100510-s001.pdf]

© 2021 Wiley-VCH GmbH

ADVANCED SCIENCE Open Access

Supporting Information

for *Adv. Sci.,* DOI: 10.1002/advs.202100510

Personalized Radiation Attenuating Materials for Gastrointestinal Mucosal Protection

*James D. Byrne, Cameron C. Young, Jacqueline N. Chu, Jennifer Pursley, Mu Xian Chen, Adam J. Wentworth, Annie Feng, Ameya R. Kirtane, Kyla A. Remillard, Cindy I. Hancox, Mandar S. Bhagwat, Nicole Machado, Tiffany Hua, Siddartha M. Tamang, Joy E. Collins, Keiko Ishida, Alison Hayward, Sarah Becker, Samantha K. Edgington, Jonathan D. Schoenfeld, William R. Jeck, Chin Hur, Giovanni Traverso**

Supporting Information

Personalized radiation attenuating materials for gastrointestinal mucosal protection

*James D. Byrne, Cameron C. Young, Jacqueline N. Chu, Jennifer Pursley, Mu Xian Chen, Adam J. Wentworth, Annie Feng, Ameya R. Kirtane, Kyla A. Remillard, Cindy I. Hancox, Mandar S. Bhagwat, Nicole Machado, Tiffany Hua, Siddartha M. Tamang, Joy E. Collins, Keiko Ishida, Alison Hayward, Sarah Becker, Samantha K. Edgington, Jonathan D. Schoenfeld, William R. Jeck, Chin Hur, Giovanni Traverso***

Supporting Figures

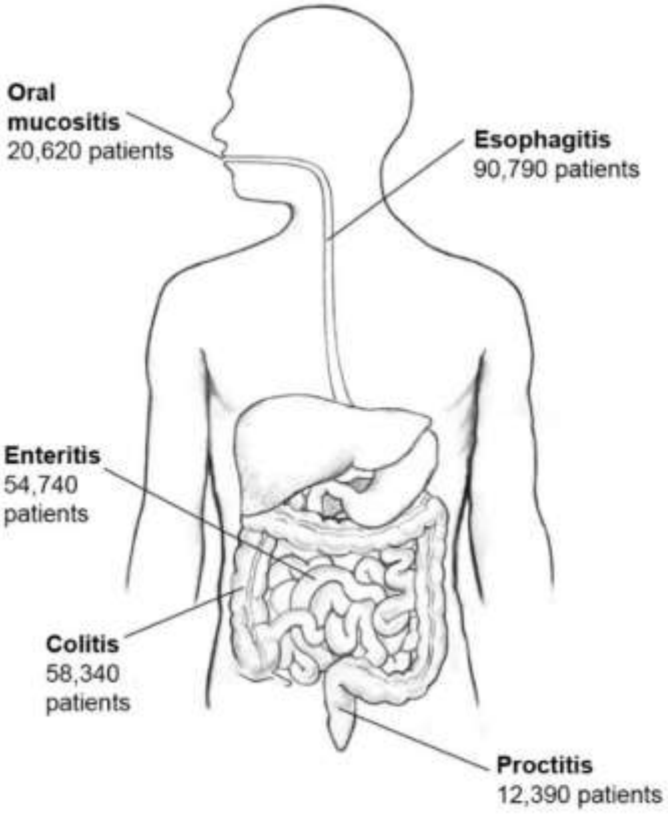

Figure S1. Estimated incidence of radiation-induced GI tissue injury.

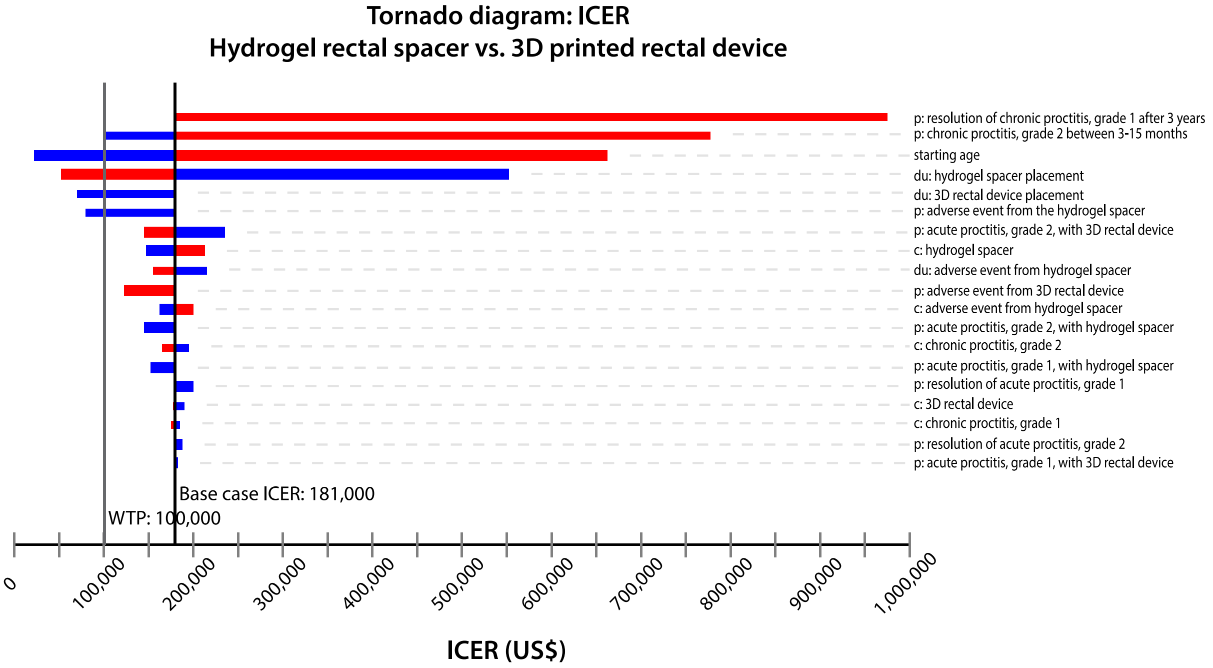

Figure S2. Tornado diagram of one-way sensitivity analyses for key parameters in the cost-effectiveness model. Parameters are listed in descending order of effect on the ICER. The base case ICER is denoted by the black line ($181,000). Changes in parameters that moved the ICER to the left of this value made the hydrogel rectal spacer more cost-effective. Changes in parameters that moved the ICER to the right of this value made the hydrogel rectal spacer less cost-effective. Changes in parameters that moved the ICER past the gray line, which denotes the WTP threshold ($100,000), made the hydrogel rectal spacer the cost-effective therapy compared to the 3D printed rectal device. Values in blue are low values of the parameter tested; values in red are high values of the parameter tested. ICER, incremental cost-effectiveness ratio; WTP, willingness-to-pay; p, probability; du, disutility; c, cost.

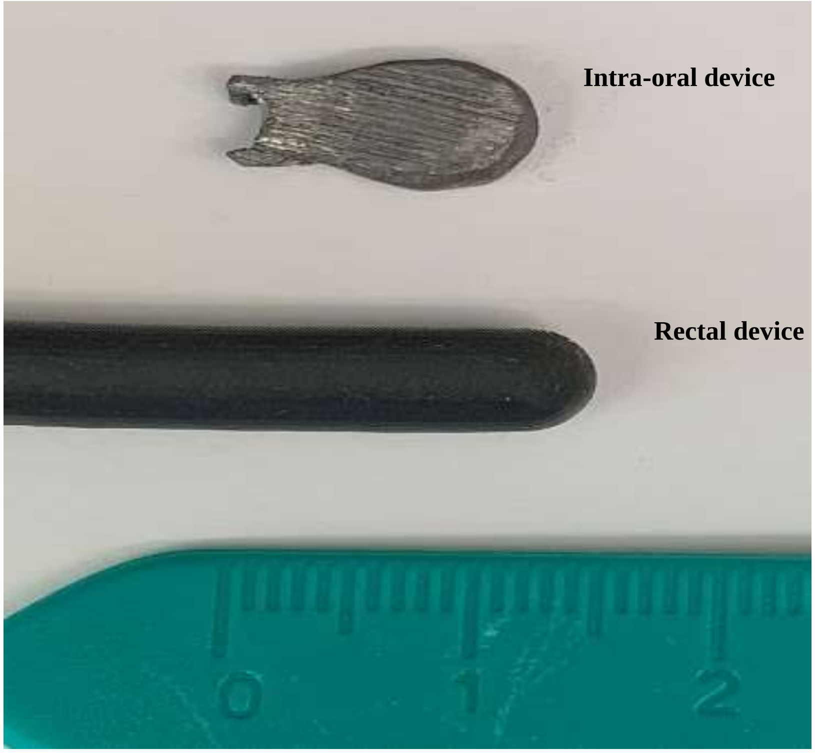

Figure S3. Devices used for single dose radiation-induced oral mucositis and proctitis rat model.

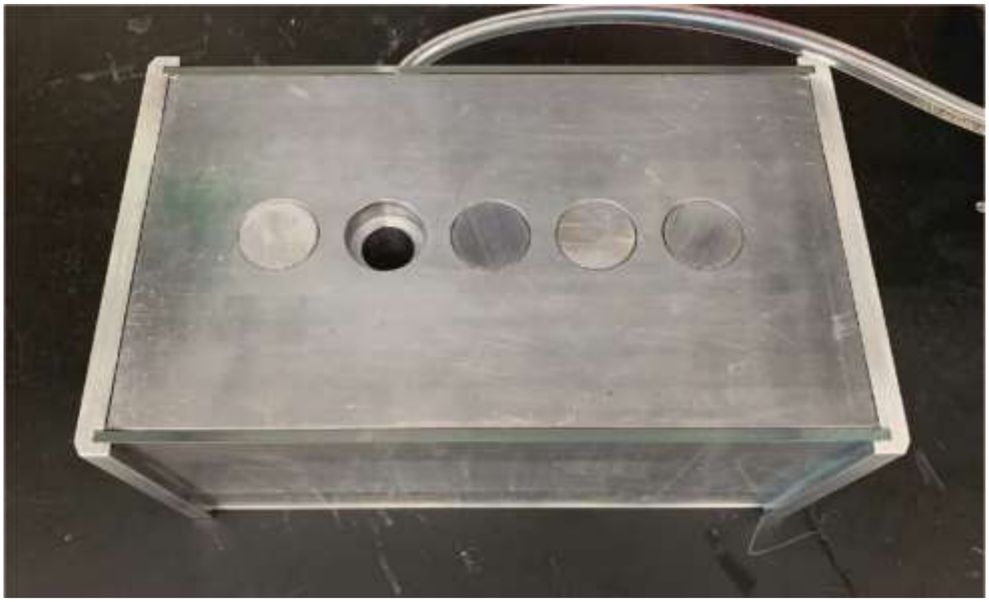

Figure S4. Customized lead irradiator shielding with 2 cm collimator used in GammaCell irradiator. Anesthesia ports are located on the sides of the shielding to maintain anesthesia.

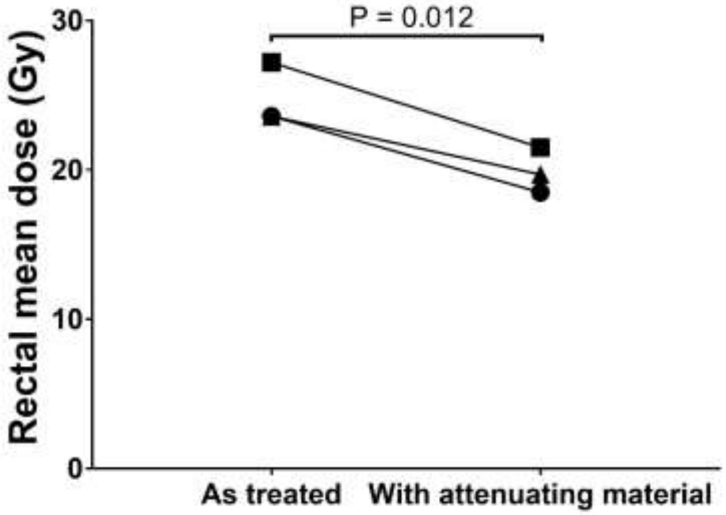

Figure S5. Dosimetric modeling of radioprotectant devices in prostate cancer patients using plan recalculations. P value was determined by paired two sample t test.

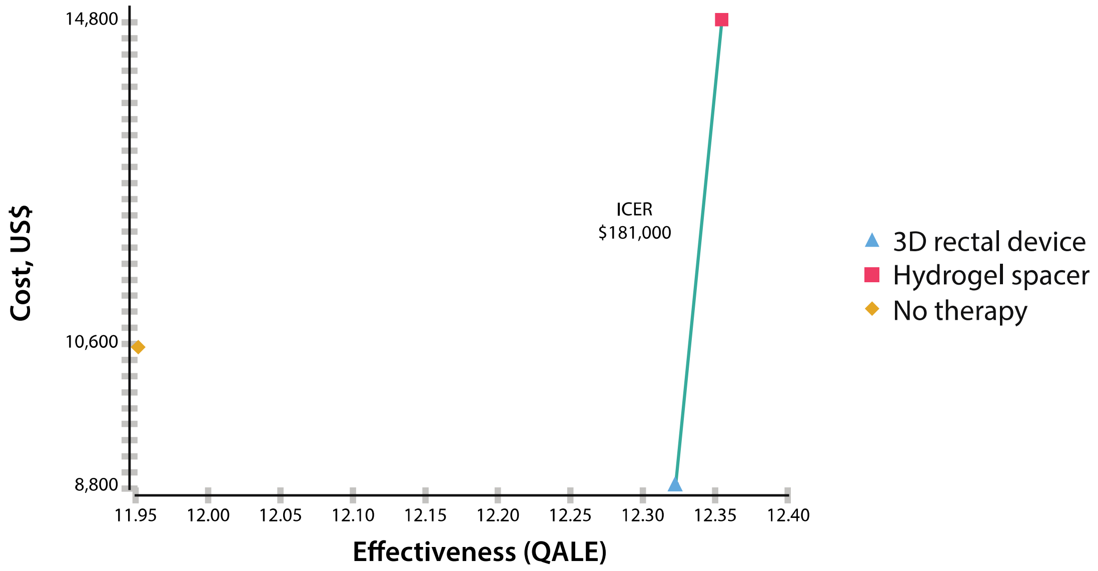

Figure S6. Efficiency frontier for the cost-effectiveness model base case analysis. The 3D printed rectal device is the cost-effective strategy. The ICER for the hydrogel spacer is $181,000 per QALY, which is higher than the willingness-to-pay threshold of $100,000 per QALY. ICER, incremental cost-effectiveness ratio; QALE, quality-adjusted life expectancy.

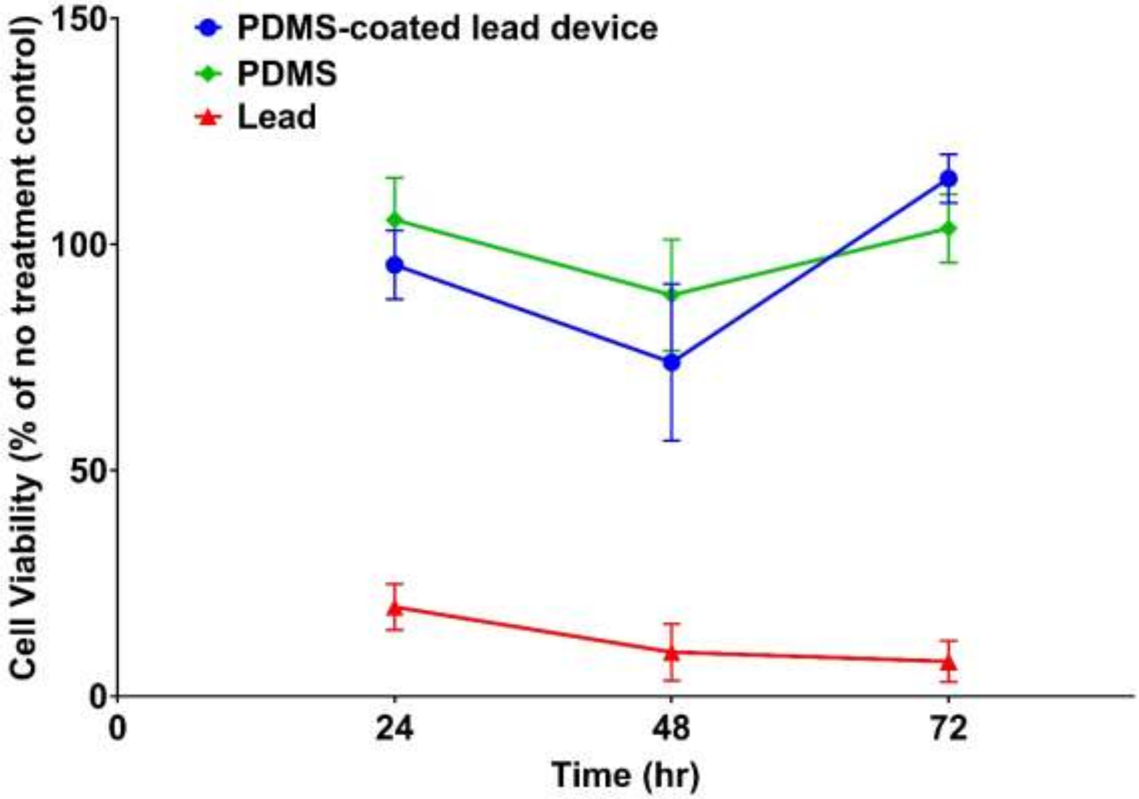

Figure S7. Cytotoxicity testing of PDMS-coated intraoral radioprotective lead device compared to controls in a human colorectal epithelial cancer cell line (n = 9 per treatment). Error bars represents mean +/- standard deviation. PDMS – polydimethylsiloxane.

Supporting Tables

Table S1. Parameters for the cost-effectiveness analysis.

Parameter	Base case	Sensitivity analysis	References
Starting age	67	30-95	14, 26, 38
Probability of death, all-cause	Variable		39-40
Probabilities			
No therapy arm			
Probability of acute proctitis, grade 1	0.278	0-0.278	14, 26, 38
Probability of acute proctitis, grade 2+	0.042	0-0.042	14, 26, 38
Probability of chronic proctitis, grade 1, from 3-15 mo	0.056		14, 26, 38
Probability of chronic proctitis, grade 2+, from 3-15 mo	0.014		14, 26, 38
Probability of chronic proctitis, grade 1, from 15-37 mo	0.09		14, 26, 38
Probability of chronic proctitis, grade 2+, from 15-37 mo	0.057		14, 26, 38
Hydrogel rectal spacer arm			
Probability of acute proctitis, grade 1	0.23	0-0.23	14, 26, 38
Probability of acute proctitis, grade 2+	0.041	0-0.041	14, 26, 38
Probability of chronic proctitis, grade 1, from 3-15 mo	0.02	0-0.005	14, 26, 38
Probability of chronic proctitis, grade 2+, from 3-15 mo	0	0-0.05	14, 26, 38
Probability of chronic proctitis, grade 1, from 15-37 mo	0.02	0-0.005	14, 26, 38
Probability of chronic proctitis, grade 2+, from 15-37 mo	0	0-0.05	14, 26, 38
Probability of adverse event from spacer	0.1	0-0.1	14, 26, 38
3D printed rectal device arm			
Probability of acute proctitis, grade 1	0.2423	0.23-0.254	estimate
Probability of acute proctitis, grade 2	0.04125	0.041-0.0415	estimate
Probability of chronic proctitis, grade 1, from 3-15 mo	0.029	0.02-0.038	estimate
Probability of chronic proctitis, grade 2+, from 3-15 mo	0.0035	0-0.007	estimate
Probability of chronic proctitis, grade 1, from 15-37 mo	0.038	0.029-0.0556	estimate
Probability of chronic proctitis, grade 2+, from 15-37 mo	0.0109	0.0035-0.0289	estimate
Probability of adverse event from 3D rectal device	0	0-0.05	estimate
All arms			
Probability of resolution of acute proctitis, grade 1	1	0.5-1	40-41
Probability of resolution of acute proctitis, grade 2	0.9	0.5-1	40-41
Probability of new chronic proctitis after 37 months	0		42
Probability of resolution of chronic proctitis, grade 1, by 3 years	0.65	0-.65	43
Probability of resolution of chronic proctitis, grade 1, after 3 years	0	0-0.05	43

Probability of resolution of chronic proctitis, grade 2, by 3 years	0.5	0-.5	43
Probability of resolution of chronic proctitis, grade 2, after 3 years	0	0-0.05	43
Costs (US$)			
Cost of grade 1 toxicity	217	0-634	44
Cost of grade 2+ toxicity	1355	0-2700	45
Cost of hyrogel spacer and placement	4237	3177-5296	44
Cost of 3D printed rectal device	300	10-375	46
Cost of adverse event from hydrogel spacer	24652	18489-30815	47
Cost of adverse event from 3d shield	24652	18489-30815	47
Cost of follow up care	629	314-1257	45
Utilities			
Well, without proctitis	1		Assumption
Grade 1 toxicity	0.9	0.12-0.98	48-49
Grade 2 toxicity	0.8	0.7-0.9	45
Disutility from acute grade 1 toxicity	-0.1	-0.02 - -0.88	48-49
Disutility from acute grade 2 toxicity	-0.2	-0.3 - -0.1	45
Disutility of adverse event from hydrogel spacer	-0.1	-0.15 - -0.05	49
Disutility from hydrogel spacer placement	-0.0785	-0.18 - -0	50-51
Disutility of adverse event from 3D rectal device	-0.1	-0.15 - -0.05	49
Disutility from 3D rectal device placement	0	-0.05 - -0	46

Table S2. Rectal angle differences between patients as contoured on CT imaging.

Patient #	Rectal Angle	Image
1	68	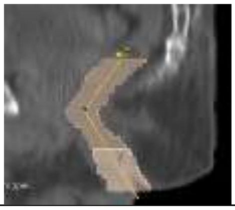
2	69	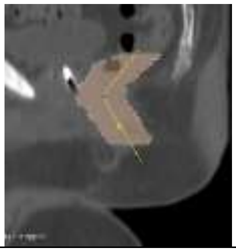
3	51	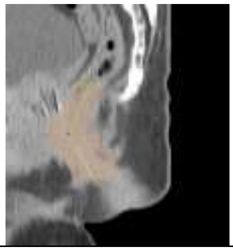
4	73	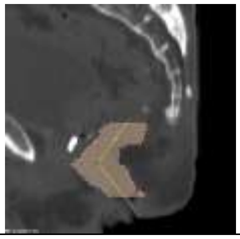
5	48	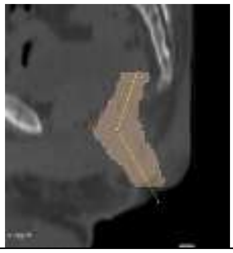
6	57	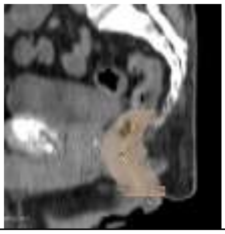

7	70	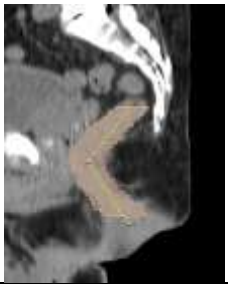
8	57	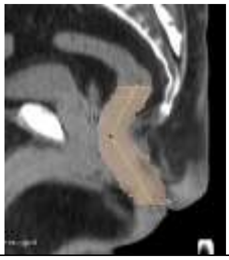
9	62	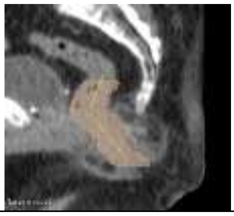
10	69	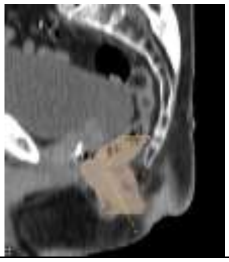
Average	62.4	

Table S3. Dose metrics for primary target and organs at risk for prostate cancer patients with and without radioprotective devices showcased in Figure 5.

Target/Organ at risk	Patient 1 – as treated	Patient 1 – with device	Patient 2 – as treated	Patient 2 – with device	Patient 3 – as treated	Patient 3 – with device
PTV – 100% of prescription dose	99.8 %	99.2 %	99.3 %	99.3 %	99.4 %	99.0 %
Bladder V65 Gy	8.1 %	7.8 %	4.2 %	3.8 %	6.5 %	5.6 %
Left femoral head V50 Gy	0 %	0 %	0 %	0 %	0 %	0 %
Right femoral head V50 Gy	0 %	0 %	0 %	0 %	0 %	0 %
Rectum – V70 Gy	7.7 %	6.2 %	6.2 %	3.6 %	4.7 %	3.9 %
Penile bulb - Mean	20.8 Gy	23.2 Gy	31.1 Gy	33.3 Gy	16.7 Gy	14.2 Gy

Table S4. Dose metrics for primary target and organs at risk for oral cavity cancer patients with and without radioprotective devices showcased in Figure 5.

Target/Organ at risk	Patient 1 – as treated	Patient 1 – with device	Patient 2 – as treated	Patient 2 – with device	Patient 3 – as treated	Patient 3 – with device
PTV: V100% Rx	97.6% (PTV56)	97.6% (PTV56)	97.5% (PTV54)	97.8% (PTV54)	94.7% (PTV60)	94.5% (PTV60)
PTV: V100% Rx	95.7% (PTV63)	96.9% (PTV63)	98.1% (PTV60)	98.1% (PTV60)	99.4% (PTV64)	99.4% (PTV64)
PTV: V100% Rx	99.3% (PTV70)	99.9% (PTV70)				
Parotid_L: V30	16.4%	16.0%	61.9%	61.7%	41.6%	41.3%
Parotid_L: Mean	23.6 Gy	23.0 Gy	39.8 Gy	39.5 Gy	29.3 Gy	28.9 Gy
Parotid_R: V30	48.3%	47.7%	21.6%	22.4%	36.1%	36.2%
Parotid_R: Mean	37.8 Gy	37.1 Gy	22.7 Gy	22.6 Gy	25.9 Gy	25.5 Gy
SMG_L: Mean	58.1 Gy	57.6 Gy				
SMG_R: Mean	69.2 Gy	69.2 Gy				
Constrictors: Mean	47.4 Gy	47.4 Gy	49.9 Gy	50.1 Gy	55.3 Gy	55.5 Gy
Mandible: Max	72.7 Gy	72.5 Gy	63.6 Gy	64.3 Gy	67.1 Gy	67.3 Gy

Table S5. Sensitivity analysis for efficacy of the rectal device.

3D rectal device - efficacy compared to hydrogel spacer	Cost (US$)	QALE	Hydrogel spacer ICER (US$/QALY)
**100%**	8,300	12.4	Dominated
**75% (base case)**	8,800	12.32	181,000

Table S6. Currently available radioprotectant technologies.

Technology	Mechanism	Use case	Degree of attenuation	Reference
Our 3D printed radioprotectant device	Shielding	Head and neck cancer	Reduction in mean buccal dose by 30% compared to no device	-
Our 3D printed radioprotectant device	Shielding	Prostate cancer	Reduction in mean rectal dose by 15% compared to rectal balloon	-
SpaceOAR	Displacement	Prostate cancer	Reduction in rectal mean dose reduction by 8.0 Gy; reduction in rectal V70 reduction by 25% compared to no device	52
ProSpace	Displacement	Prostate cancer	Reduction in the rectal V70% by 55% compared to no device	53
Clamshell	Shielding	Testicular cancer	Reduction in mean testicular dose by 59% compared to no device	54
Eye shield (tungsten)	Shielding	Ocular/periocular lesions	Reduction in mean lens dose by 96% compared to no device	55
Eye shield (bismuth)	Shielding	Ocular/periocular lesions	Reduction in mean lens dose by 50% compared to no device	56
Mouth guard	Displacement	Head and neck cancer	-	57
Rectal balloon	Displacement	Prostate cancer	Reduction in rectal V70 by 38.5% compared to no device	31
Leaded apron	Shielding	Multiple	-	59
Leaded thyroid shield	Shielding	Multiple	Reduction in mean thyroid by 48% compared to no device	59
